# Endothelium as a Source and Target of H_2_S to Improve Its Trophism and Function

**DOI:** 10.3390/antiox10030486

**Published:** 2021-03-19

**Authors:** Valerio Ciccone, Shirley Genah, Lucia Morbidelli

**Affiliations:** Department of Life Sciences, University of Siena, Via A. Moro 2, 53100 Siena, Italy; ciccone3@student.unisi.it (V.C.); shirley.genah@student.unisi.it (S.G.)

**Keywords:** vascular endothelium, hydrogen sulfide, endothelial dysfunction, hypertension, atherosclerosis, diabetes, angiogenesis, wound healing, H_2_S donors

## Abstract

The vascular endothelium consists of a single layer of squamous endothelial cells (ECs) lining the inner surface of blood vessels. Nowadays, it is no longer considered as a simple barrier between the blood and vessel wall, but a central hub to control blood flow homeostasis and fulfill tissue metabolic demands by furnishing oxygen and nutrients. The endothelium regulates the proper functioning of vessels and microcirculation, in terms of tone control, blood fluidity, and fine tuning of inflammatory and redox reactions within the vessel wall and in surrounding tissues. This multiplicity of effects is due to the ability of ECs to produce, process, and release key modulators. Among these, gasotransmitters such as nitric oxide (NO) and hydrogen sulfide (H_2_S) are very active molecules constitutively produced by endotheliocytes for the maintenance and control of vascular physiological functions, while their impairment is responsible for endothelial dysfunction and cardiovascular disorders such as hypertension, atherosclerosis, and impaired wound healing and vascularization due to diabetes, infections, and ischemia. Upregulation of H_2_S producing enzymes and administration of H_2_S donors can be considered as innovative therapeutic approaches to improve EC biology and function, to revert endothelial dysfunction or to prevent cardiovascular disease progression. This review will focus on the beneficial autocrine/paracrine properties of H_2_S on ECs and the state of the art on H_2_S potentiating drugs and tools.

## 1. Vascular Endothelium

The vascular endothelium is the tissue that lines the inside of the circulatory system (blood vessels, lymphatic vessels and heart). The cells, arranged in a single layer oriented on the longitudinal axis of the vessel, assume a flattened shape and lay side by side with each other to form a complete monolayer. Structurally, the endothelial cell (EC) apical domain is in direct contact with blood or lymph, while the basolateral domain anchors to the basal lamina, which connects EC to the underlying tissues, such as the medial or muscular layer and the adventitia, rich in fibrous tissue.

The vascular endothelium acts as a selectively permeable barrier between extravascular and intravascular compartments and provides a non-thrombogenic surface for the cardiovascular system [1]. Nowadays, the endothelium can no longer be considered a passive barrier. Indeed, its anatomical position allows it to integrate the physical and neurohumoral signals from the blood and surrounding tissues for regulating vascular tone and permeability, cell adhesion, inflammation, smooth muscle phenotype and proliferation, as well as thromboresistance and blood fluidity [2,3].

The endothelial lining represents a wide area for the exchanges between blood and tissues (about 350 m^2^ in humans) [3]. Electron microscopy observations reveal the continuous nature of arterial endothelium, characterized by tight junctions among adjacent cells in order to limit macromolecule exchange, and by a complex micro-vesicular system involved in macromolecular transport. In spite of its apparent morphological lack of complexity, the endothelium is characterized by heterogeneity, with differences in permeability, reactivity, and biosynthesis in relation to the type of vascular district and organ considered [1,2,3].

## 2. Role of Endothelium in Physiology

The endothelium’s role as a semipermeable barrier is one of its fundamental and basic functions: it regulates macromolecule transport between the lumen and vascular smooth muscle tissue [4]. Several mechanisms control the passage of macromolecules across the endothelial barrier: (i) through ECs themselves (transcellular flux); (ii) through the cell–cell junctions (paracellular flux); and (iii) via vesicular transport.

Most biological transmitters consist of large molecules with anionic and hydrophilic features, unable to diffuse across the membrane bilayer. The majority of those transmitters are believed to move through intracellular junctions between cells, or via vesicular transport, thanks to the formation of transient channels resulting from vesicle fusion. The reorganization of the intercellular junctions, which involves actin and myosin reconfiguration or direct collapse of junctional connections, appears to be the main process by which ECs increase their permeability to solutes and water [1].

The function of the endothelium is not limited to the internal surface lining of vessels or to constitute the vascular wall in the microcirculation, but it produces and releases vasoactive factors such as nitric oxide (NO), prostacyclin (PGI2), hydrogen sulfide (H_2_S) and endothelin (ET) which, in the appropriate concentration and balance, maintain adequate vascular tone and blood fluidity, giving the endothelium itself an antithrombotic phenotype [5,6].

The synthesis of NO by ECs is constitutive, but it can be augmented by a wide variety of compounds, including acetylcholine, angiotensin II (AngII), bradykinin, histamine, arachidonic acid. NO has a very short half-life and is synthesized from l-arginine and oxygen by the NO synthase enzyme (NOS). The endothelial isoform of this enzyme, eNOS, constitutively expressed, appears to be Ca^2+^/calmodulin-dependent. Once synthesized, NO rapidly spreads to vascular smooth muscle cells where it stimulates soluble guanylate cyclase (sGC), with an increase in cGMP formation and consequent vascular relaxation, while its autocrine function is related to the control of EC trophism and angiogenesis [7].

NO is not the only endothelial-dependent vasodilator. The endothelium also constitutively generates PGI2, which relaxes the underlying smooth muscle cells by activating adenylate cyclase and increasing cAMP. PGI2 is released in high quantities following the binding of transmitters, such as thrombin, histamine, serotonin, on cell surface receptors. The endothelium also produces and releases a hyperpolarizing factor (EDHF) whose chemical nature is still debated. EDHF’s function is to hyperpolarize vascular smooth muscle cells, causing these cells to relax and allowing dilation of blood vessels [6].

In addition to NO and carbon monoxide (CO), H_2_S is an endogenous gasotransmitter involved in the regulation of the cardiovascular, nervous, gastrointestinal, and renal systems, with a great impact on inflammatory and immune responses [8]. Recently, it has been proposed as one of the EDHFs. H_2_S exerts a multitude of physiological effects on the wall of the vessels, acting in an autocrine/paracrine manner. It is produced by vascular cells and exhibits antioxidant, anti-apoptotic, anti-inflammatory and vasoactive properties. Indeed, it reduces arterial blood pressure, limits the formation of atheromatous plaques, and promotes the vascularization of ischemic/injured tissues [8].

In some pathophysiological circumstances including hypoxia, tissue hypoperfusion or arterial hypertension, some vasoconstrictor factors may be released from the endothelium as endothelins (ETs), thromboxane A2, and prostaglandin H2. Moreover, vascular endothelium participates at the regulation of vessel tone and trophism and blood flow through the processing of angiotensin, via the expression of angiotensin converting enzyme isoforms ACE-1 and ACE-2, responsible for the balance between AngII/AT-1 receptor and Ang(1–7)/Mas receptor [9].

ECs respond to the increase in blood flow through the release of NO and PGI2 by the same cells. Indeed, shear stress causes rapid activation of eNOS and increases its gene transcription; it also endorses ECs to release factors that inhibit coagulation, leukocyte migration, and smooth muscle cell proliferation, simultaneously promoting EC survival. Conversely, low shear stress and turbulent blood flow promote a pathological feature in the endothelium responsible for atherosclerosis ignition, documenting the pivotal role of ECs in finely controlling vascular functions [2,6].

A healthy functioning endothelium also provides protection against radical species of oxygen and nitrogen (ROS/RNS). It is now clear that increased levels of ROS and RNS are harmful to cells and tissues and are involved in a wide range of cardiovascular diseases having endothelial dysfunction as an underlying phenomenon. This boosted the concept of oxidation as synonymous with cell damage and senescence. The post-translational modifications involving RNS share a common ancestor—high NO concentrations mainly synthetized by inducible NOS (iNOS), upregulated in response to various endotoxin or cytokine signals. Several pathological states are linked to the deregulation of NO levels, indicating that aberrant production of NO and its products can have deleterious consequences on cells [10]. Again, one of the functions related to a healthy endothelium is the scavenging activity of reactive species through the production of antioxidant products as H_2_S or protective enzymatic pathways.

The intimate surface of a healthy endothelium is both anticoagulant and antithrombotic: ECs secrete a wide range of molecules relevant to the regulation of blood clotting and platelet functions, as PGI2 and NO. Damage to the vessels or exposure to certain cytokines and proinflammatory stimuli overturns the equilibrium towards a procoagulant and prothrombotic EC phenotype, through the exposure of basal membrane components and/or tissue factor, and reduced presence of glycosaminoglycans or tissue factor inhibitor [5].

Endothelial trophism is guaranteed by the response to vasoactive and growth factors produced by surrounding tissues or autocrinally by the same ECs. Among the various examples, we and others have contributed to characterizing the beneficial effects on vascular endothelium by NO derived from eNOS, bradykinin, substance P, vascular endothelial growth factor (VEGF), fibroblast growth factor-2 (FGF-2), prostaglandin E2, H_2_S [11,12,13,14,15]. The molecular mechanisms responsible for cell survival, proliferation, migration and functioning include eNOS/NO/cGMP/protein kinase G (PKG), PI-3K/Akt, MAPK/ERK1/2 and gene transcription of autocrine factors as FGF-2 [7].

Epigenetics is an emergent mechanism involved in the regulation of vascular biology and endothelial trophism. Through chromatin structure modification, epigenetics can modify endothelial functions with an impact on cardiovascular disease, being the regulatory functions of epigenetics also active on endothelial precursor cells and circulating angiogenic cells [16,17]. DNA methylation, variants, histone post-translational modifications, and recently discovered RNA-based mechanisms represent the major pathways involved in the molecular basis of epigenetics. VEGF-A and NOS are the key players in regulating and maintaining cardiovascular functions. Their expression can be controlled by epigenetic mechanisms. In particular, VEGF-A epigenetic control can occur mainly through changes in histone code by RNAs. VEGF-A acts through VEGFR2, which in turn is regulated by promoter DNA methylation [18]. Furthermore, accumulating evidence indicates that epigenetic pathways play an important role in eNOS gene regulation [19].

These findings suggest the importance to deeply understand the epigenetic mechanisms involved in the regulation of vascular functions both in physiology and in pathological conditions.

## 3. Endothelial Dysfunction

Endothelial dysfunction refers to a systemic condition in which the endothelium loses its physiological properties, including the control of vasodilation, fibrinolysis and platelet aggregation. Key features of the endothelial dysfunction are: (1) the reduced local production of NO due to impaired activity (uncoupling) of eNOS, and of other vasodilating mediators as H_2_S; (2) the decrease in anticoagulant factors such as heparin; (3) the increase in the secretion of reactive species, von Willebrand factor, and tissue factor; (4) the overexpression of adhesion molecules for leukocytes and platelets [20,21]. All these factors concur to compromise the physiological vascular homeostasis. Due to the impairment of the main protective transmitters NO and H_2_S, the resulting endothelial dysfunction is associated with increased ROS and RNS levels and vascular oxidative stress [22,23]. The perpetuation of this condition then leads to retraction and death of the endothelium with increased permeability and exposure of the components of the basement membrane, that further amplifies the picture of vascular inflammation [4,20,22].

Endothelial dysfunction risk factors are represented by pathological states such as hypertension, diabetes, and hyperlipidemia, and improper lifestyles such as high-fat diets, tobacco and alcohol consumption, and physical inactivity [24]. Most cardiovascular diseases share endothelial dysfunction as a hallmark: atherosclerosis, diabetes complications, thrombosis, and hypercoagulation [20]. Moreover, physiological ageing through the phenomenon of mild chronic inflammation (“inflammaging”) is accompanied by endothelial dysfunction [25]. Inflammatory factors such as tumor necrosis factor-α (TNF-α), inteleukin-6 (IL-6), intercellular adhesion molecule 1 (ICAM-1), and loss of the antioxidant mechanism are among the most influential promoters of vascular impairment [21].

Mounting evidence suggests that epigenetic mechanisms may contribute to vascular complications in many pathological conditions, such as diabetes or atherosclerosis, linked to altered endothelial trophism and functions [17,26]. The inflammatory phenotype in ECs induces the transcription of many cytokines and adhesion molecules, in a nuclear factor-kappa B (NF-κB)-dependent manner. Epigenetic modifications in the NF-κB promoter region produce an increased expression of p65 subunit of NF-κB, and a hyper-activation of the NF-κB pathway [27]. Another mechanism could involve histone deacetylase 2 (HDAC2), which interacts and deacetylates Nrf2. Oxidized low-density lipoproteins (ox-LDLs) are able to downregulate HDAC2 expression, resulting in increased production of eNOS-dependent reactive species [16]. Furthermore, shear stress represented by blood flow alteration modifies EC gene expression and function. Dunn et al. demonstrated that disturbed blood flow stimulates DNA methyltransferase-1 (DNMT-1) expression in endothelial cells with aberrant DNA methylation at the promoter of flow-inducible genes, contributing to atherosclerosis [28]. Finally, recent data showed the role of non-coding RNA in regulating the expression of endothelial adhesion molecule [29].

Acute or chronic infections both by bacteria and viruses have cardiovascular consequences for their direct or indirect effects on vascular endothelium, through bacterial products or cytokines released by tissue and immune system cells [30,31], and through epigenetic regulation [32]. The recent pandemic due to SARS-CoV-2 supports this concept [33,34].

Furthermore, our studies and those of others have revealed that endothelial dysfunction is associated to impaired EC survival and physiological angiogenic outcomes with subsequent rearrangement of the microcirculation that contributes to the emergence of various pathological conditions and healing disorders [7,35]. ECs play a key role in the adaptation of tissues to damage, revealing their plasticity. A change in endothelial functions following ischemia can induce the transition to a mesenchymal phenotype characterized by functional, metabolic and gene expression signatures. Indeed, the mesenchymal phenotype, with increased cell migration and clonal expansion, participates in regenerating a functioning vascular network [36].

Considering endothelial function as a “barometer for cardiovascular risk”, it is crucial to identify the molecular determinants underlying endothelial integrity and functionality. Seeing endothelium as an exchange regulator between the vascular wall and surrounding tissues, it is expected that dysfunctional ECs can determine damage to other tissues [37]. Indeed, a detailed assessment of the cellular and molecular mechanisms at the base of vascular function, and, particularly, of endothelial dysfunction, will help the diagnosis and treatment choice for a broad array of human disorders, including cardiovascular and neurodegenerative diseases [21,24,38,39,40].

## 4. Biochemistry of H_2_S Production

H_2_S is a gas physiologically produced by tissue and vascular cells. The enzymes responsible for the synthesis of H_2_S are cystathionine β-synthase (CBS), cystathionine-γ-lyase (CSE), and 3-mercaptopyruvate sulfurtransferase (3-MST). The first two enzymes use l-cysteine as a substrate and are dependent on pyridoxal-5′-phosphate. 3-MST, on the other hand, works in association with cysteine aminotransferase which, starting from l-cysteine and α-ketoglutarate, produces 3-mercaptopyruvate. These enzymes are differentially expressed in the various tissues. In particular, the expression of CSE and 3-MST predominates in the cardiovascular system [8,41]. CSE is only present in the cytoplasm, while CBS and 3-MST both have a cytosolic and mitochondrial form, with the latter predominating. CSE is the principal enzyme responsible for H_2_S biosynthesis, located in vascular smooth muscle cells and found mainly in ECs [42,43]. This can explain why the concentration of H_2_S in the vascular tissues is around 100 times greater than in other tissues [44], suggesting a crucial role in vascular homeostasis, endothelial function, and trophism [45]. In addition, H_2_S can be also generated via a reduction in thiols and thiol-containing molecules, in a non-enzymatic manner [8]. Compared to CBS, knockout mice for CSE have no severe phenotype and normal lifespan. Their phenotype is mainly cardiovascular, with hypertension and endothelial dysfunction [46].

**Figure 1:**
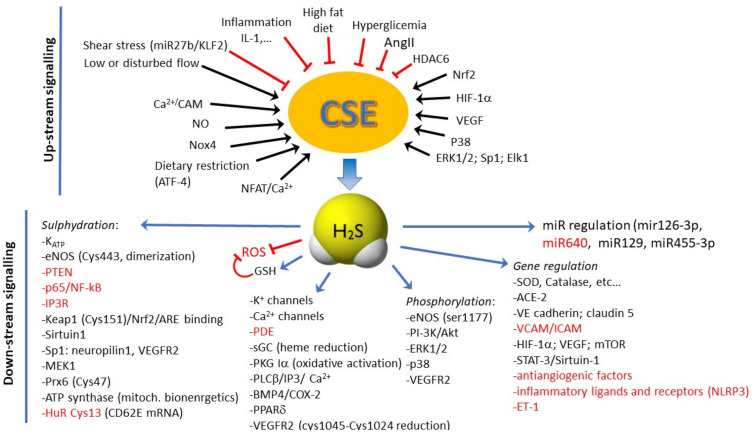
Summary of the molecular mechanisms controlling CSE expression and function in endothelial cells (up-stream signaling) and of the multiple downstream signaling activated or inhibited by H_2_S in ECs. Red target or lines means inhibition. Note that some signals are both up- and downstream, strengthening the central role of CSE/H_2_S in controlling vascular trophism and functions. ACE-2, angiotensin converting enzyme 2; Akt, protein kinase B; AngII, angiotensin II; ARE, antioxidant responsive elements; ATF4, activating transcription factor 4; BMP4, bone morphogenetic protein 4; cGMP, cyclic guanosine monophosphate; COX-2, cyclooxygenase-2; CSE, cystathionine γ-lyase; Elk1, ETS Like-1 protein; eNOS, endothelial NO synthase; ERK1/2, extracellular signaling regulated kinase ½; ET-1, endothelin-1; HIF-1a, hypoxia inducible factor-1a; HuR, human antigen R; ICAM, intercellular adhesion molecule; IL-1, interleukin 1; IP3, inositol-3-phosphate; IP3R, inositol-3-phosphate receptor; K_ATP_, ATP-sensitive K+ channels; Keap1, Kelch-like ECH associated protein 1, KLF2, Krüppel-like Factor 2; MAPK, mitogen-activated protein kinase; MEK1, MAP kinase kinase 1; NFAT, nuclear factor of activated T-cells; NF-κB, nuclear factor-kappa B; NLRP3, nucleotide-binding oligomerization domain, leucine rich repeat, and pyrin domain containing protein 3; NO, nitric oxide; Nox4, NADPH oxidase 4; Nrf2, nuclear factor erythroid 2-related factor 2; p38, p38 mitogen-activated protein kinases; PDE, phosphodiesterase; PI-3K, phosphoinositide 3-kinase; PKG, protein kinase G; PLCβ, phospholipase Cβ; PPARδ, peroxisome proliferators-activated receptor δ; Prx6, thioredoxin-dependent peroxiredoxin; PTEN, phosphatase and tensin homolog; sGC, soluble guanylate cyclase; SOD, superoxide dismutase; Sp1, specificity protein 1 transcription factor; STAT3, signal transducer and activator of transcription 3; VCAM, vascular cell adhesion molecule; VEGF, vascular endothelial growth factor; VEGFR2, vascular endothelial growth factor receptor 2.

Information has become available about the regulation of the expression and activity of these enzymes (Figure 1). It has been reported that NADPHox4 derived ROS (via heme-regulated inhibitor kinase/eIF2/activating transcription factor 4 (ATF4) signaling) enhance the expression of CSE [47]. Blood flow has been reported to exert divergent effect of H_2_S producing enzymes, depending on the type of endothelium and flow stress. While laminar flow was initially demonstrated to enhance the expression of CSE and 3-MST in ECs [47,48], recent data are more complex. In particular, laminar flow (high shear stress) has been demonstrated to inhibit CSE expression via KLF2 regulated miRNA-27b [49], while turbulent flow (low shear stress) seems to upregulate CSE [50,51]. In rat aortic ECs, calcium-sensing receptors increase CSE expression in a phospho-calmodulin kinases II-dependent manner to inhibit platelet activation [52]. Transcription factors specifically controlling CSE expression are among the others NF-κB in lipopolysaccharide (LPS)-stimulated macrophages [53], specificity protein 1 (Sp1) in smooth muscle cells [54] and Elk1 in beta pancreatic cells [55]. Nuclear factor of activated T cell (NFAT) binding sites have been identified in the CSE promoter. Intermittent hypoxia exposure reduces Ca^2+^-dependent activation of calcineurin/NFAT to lower CSE expression and impair vasodilation, while NFAT activation lowers CSE expression at the cell and microvascular levels [56].

OxLDL in one of the major stimuli to cause the endothelial damage that leads to atherosclerosis. One of the epigenetic mechanisms underlying CSE reduced expression at both mRNA and protein levels by oxLDL is increased histone deacetylase 6 (HDAC6) [57]. Recently this finding has been extended to blood pressure control, by assessing the role of tubastain A in AngII induce hypertension, and documenting that upregulation of CSE and H_2_S through HDAC6 inhibition can be a valid therapeutic strategy [58].

Additional information on CSE gene and enzyme control is provided in the chapters below and is summarized in Figure 1.

H_2_S plasma levels are kept at appropriate concentrations by three elimination systems. The first pathway involves mitochondrial oxidative metabolism which converts H_2_S into thiosulfate, followed by further conversion into sulfate and then sulfite. The second metabolic pathway is cytosolic methylation to dimethyl sulfide via thiol S-methyltransferase. Finally, the binding of H_2_S to hemoglobin leads to the formation of sulfhemoglobin [45].

## 5. Molecular Signaling Activated by H_2_S into ECs

H_2_S in ECs performs a protective action on vessels in an autocrine/paracrine manner. It plays a role in the regulation of vasodilation, angiogenesis, inflammation, oxidative stress and apoptosis [59]. Three are the main mechanisms through which H_2_S exerts it biological effect: (i) reactive oxygen species/nitrogen species scavenging; (ii) interaction with metal centers; (iii) persulfidation (called also S-sulfhydration).

H_2_S acts through a post-translational modification—the S-sulfhydration of cysteine residues, which modifies the structure and activity of the target proteins [60]. The mechanism is persulfidation on reactive cysteine residues (-SH) of target proteins to form a persulfide group (-SSH). An example is the persulfidation of the ATP-dependent K channel (K_ATP_) in ECs and smooth muscle cells, responsible for fast hyperpolarization and vasorelaxation [61]. Indeed, evidence has been provided in support of H_2_S function as an EDHF, exerting more remarkable vasorelaxation in the peripheral resistance arteries [62].

H_2_S also reduces ROS levels through their direct inactivation and by enhancing antioxidant defense mechanisms. One of the mechanisms underlying oxidative protection is given by the H_2_S regulation of the Keap1/Nrf2 pathway. Normally, the transcription factor Nrf2 is inhibited by its binding to Keap1 in the cytoplasm. In conditions of oxidative stress, H_2_S promotes the translocation of Nrf2 into the nucleus by means of the S-sulfhydration of the Keap1 inhibitor, causing the dissociation of the Keap1/Nrf2 complex. Nrf2 in the nucleus activates the antioxidant responsive element. Consequently, the transcription of many antioxidant genes such as superoxide dismutase, catalase, glutathione peroxidase and glutathione-S-transferase is induced, requiring hours or days to produce a biological effect [63].

Among other targets, H_2_S decreases inflammation by inhibiting transcription factors such as NF-κB through persulfidation [64], thus decreasing the expression of pro-inflammatory mediators. Most persulfidation reactions lead to target inhibition as phosphatase and tensin homolog (PTEN) [65], except for MEK1 activity, which in HUVEC leads to increased activity with DNA damage repair and senescence impairment [66]. Recently, Prx6 has been identified as a further target of sulfhydration on Cys47, which controls its decamerization and peroxidase activity [49], while the “S-sulphydrome” was identified among the many target proteins, and β3 integrin was identified as the key element of endothelial mechanotransduction [67].

The interaction of H_2_S with the NO/NOS pathway involves different modalities: as inhibition of PDE in smooth muscle cells, PI-3K/Akt-dependent phosphorylation of eNOS in Ser1177 [68] and stabilization of eNOS in the dimeric state through enzyme persulfidation [69]. Additionally, heme reduction in sGC enzyme with facilitated response to NO [70] and activation of protein kinase G Iα (PKG Iα) through disulfide bond formation [71] have been reported to potentiate the NO/cGMP pathway.

H_2_S also stimulates endothelial proliferation and migration, aiding the process of angiogenesis and wound repair. H_2_S acts at several levels on the mechanisms responsible for angiogenesis, including the control of VEGF expression, through upregulation of the transcription factor hypoxia inducible factor-1α (HIF-1α) or direct modulation of the PI-3K and Akt pathways in ECs (signaling pathways also activated by VEGF) [41,72,73]. In angiogenic ECs, H_2_S has also been reported to activate signal transducer and activator of transcription 3 (STAT3) [74], mammalian target of rapamycin (mTOR) and the VEGFR2 pathway [75]. It has been reported that CBS silencing in ECs reduces VEGF signaling through VEGFR2 and neuropilin-1 downregulation [76].

A schematic summary of the molecular mechanisms activated or inhibited by H_2_S in ECs is reported in Figure 1. As a note, the effects of H_2_S are context and tissue-dependent, sometimes producing divergent functional effects. It is plausible that this depends on endothelial heterogeneity, tissue microenvironment, and physio-pathological conditions where there is the influence of epigenetic mechanisms.

## 6. Cardiovascular Diseases Associated with Altered Levels of H_2_S

### 6.1. Hypertension

Altered levels of H_2_S have been reported in both experimental models and clinical studies on patients with severe hypertension, where lower plasma H_2_S levels are described along with reduced content of CBS and CSE (see [77,78] for recent reviews). A human cohort study demonstrated a reduced H_2_S plasma level in hypertensive patients, suggesting H_2_S as a potential therapeutic target and diagnostic marker [79].

The intimate relationship between H_2_S-associated endothelial dysfunction and hypertension comes from the observation that CSE KO mice develop hypertension particularly with impaired endothelium-dependent relaxation in resistance mesenteric arteries [46].

The levels of H_2_S-producing enzymes are reduced in the vessel wall of spontaneous or drug induced hypertensive animals [80,81,82]. Exposure of cultured endothelial cells to AngII and, similarly, to H_2_O_2_, downregulated the expression and activity of CSE with induction of endoplasmic reticulum stress [83]. In a mouse model of Ang II-induced hypertension, H_2_S reversed the aortic endothelial dysfunction and reduced NO bioavailability, while blockade of endogenous H_2_S exacerbated these alterations [84]. Other studies demonstrated that administration of H_2_S donors decreases blood pressure and reverses vascular remodeling through the suppression of smooth muscle cell proliferation and collagen deposition in the vessel wall [61,85,86,87,88,89]. H_2_S treatment noticeably reestablishes eNOS function and NO bioavailability in N^ω^-nitro-l-arginine methyl ester (L-NAME)-induced hypertensive rats [90]. From a mechanistic point of view, H_2_S improves endothelial function through the inhibition of oxidative stress, suppression of renin angiotensin system, downregulation of bone morphogenic protein 4/cyclooxygenase-2 (BMP4/COX-2) pathway, or activation of the PPARδ/PI-3K/Akt/AMPK/eNOS cascade, thus contributing to the antihypertensive mechanism of H_2_S in renovascular hypertensive rats [91,92,93]. In SHR, administration of H_2_S significantly decreases blood pressure and abrogates endothelial dysfunction through inactivation of NLRP3 inflammasome and oxidative stress [94]. In another disease model, lead-induced hypertension in rats, H_2_S treatment normalizes blood pressure and ameliorates endothelial dysfunction with an inhibition of oxidative stress [95].

Recently, it has been proposed that H_2_S can regulate EC pathological behavior through epigenetic mechanisms. H_2_S induces miR-129, which inhibits DNA methyltransferase-3 (DNMT3) and IL-17, found to be overexpressed in hypertension [96].

Summarizing all results, it can be concluded that the CSE/H_2_S signaling pathway may represent a potential therapeutic target for hypertension.

### 6.2. Diabetes

The relation between diabetes-induced endothelial dysfunction and H_2_S impairment is now well established (for review see [77,97]). H_2_S levels have been observed to be reduced in rats with diabetes induced by streptozotocin (STZ) and in subjects with type 2 diabetes mellitus [98,99,100,101,102]. In line with these findings, a high fat diet downregulates and dietary restriction induces (via ATF4) CSE expression [103,104]. Hyperglycemia lowers H_2_S levels due to the high H_2_S catabolism favored by the extremely oxidizing environment or the reduced gasotransmitter production due to a lower expression of the generating enzymes or their inhibition such as oxidative inactivation of the 3-MST at endothelial level [105]. The lack of H_2_S bioavailability supports the accumulation of intracellular ROS, which are not completely scavenged by H_2_S due to its consumption in high-glucose-treated ECs [106]. The consequent oxidative status favors mitochondrial dysfunction and mitophagy, cell damage, and apoptosis [107,108,109,110].

Endothelial dysfunction in diabetes correlates with angiogenesis impairment. CSE expression and H_2_S levels are strongly diminished in wound granulation tissues of obese diabetic mice [111], thus explaining the angiogenesis impairment described in wounds and critical limb ischemia in diabetes [112,113,114]. The availability of H_2_S donors or CSE upregulators could be an innovative therapeutic strategy to promote endothelial function and proper neovascularization of wounds. However, despite the protective effect of H_2_S on endothelial function and wellness, the stimulation of angiogenesis in atherosclerosis plaques by high CSE expression could have a negative outcome, resulting in plaque vulnerability and rupture [115]. The choice of the proper strategy and best control of H_2_S at tissue level is still a critical point to be resolved at the experimental and clinical level.

## 7. Molecular Mechanisms Regulated by H_2_S in Support of EC Function and Trophism

### 7.1. Antioxidant and Anti-Inflammatory Properties

Several studies document that H_2_S limits vascular permeability, directly or indirectly through antioxidant and anti-inflammatory actions. The multiplicity of the mechanism downstream H_2_S production is schematically reported in Figure 1. Vascular hyperpermeability was inhibited in mice undergoing cardiac arrest and blood–brain barrier disruption following H_2_S inhalation [116]. This protective effect was linked to reduced expression of VEGF and metalloproteinase-9 and increased angiopoietin-1. Another study documented scavenging of ROS and activation of Akt [117]. Data, however, are not all in the same direction, documenting that the final effect is context dependent.

The protective effect of H_2_S in conditions such as hypertension, atherosclerosis, and vascular diabetic complications may be related to multiple actions by the gasotransmitter: H_2_S inhibition of ROS production, blunting of ROS by direct scavenging, upregulation of glutathione, and antioxidant enzymes [101,118]. H_2_S reduces ROS levels in ECs exposed to high glucose, preventing their apoptosis and damage [108,119]. Gene transfer of CSE or administration of exogenous H_2_S in diabetes models reduced ROS levels and improved endothelial dependent vasorelaxation, while CSE KO was responsible for a greater impairment of endothelial function [106]. Many studies support the inhibitory effect of H_2_S on endothelial inflammation [120]. The autocrine/paracrine action of endothelial-derived H_2_S has been documented by endothelial specific deletion of CSE, which predisposes to vascular inflammation and atherosclerosis [50].

In ECs exposed to high glucose, a suppression of NF-κB activity and reduction in ICAM-1 levels were found upon NaHS pretreatment [121]. Moreover, stimulation of ECs with high glucose significantly promotes ET-1 secretion, which was reduced by administration of H_2_S [122].

Recently, inhibition of necroptosis together with ROS downregulation have been described in ECs exposed to hyperglycemia [123]. Inhibition of adhesion molecules such as ICAM-1 in ECs has been described in response to NaHS through NF-κB inhibition [124], while CSE inhibition increased leukocyte adherence to the endothelium [125]. The anti-inflammatory activity of H_2_S is not only related to the impairment of adhesion molecules as vascular cell adhesion molecule (VCAM) and ICAM, but also to the inhibition of inflammatory mediator production, such as IL1-β, TNF-α, IL-6 and monocyte chemoattractant protein-1 by ECs and monocytes/macrophages [126,127]. IL-1β, in turn, was found to be increased in atherosclerotic plaques, and induces the phosphorylation of Ser377 and inactivation of CSE [127].

In cultured ECs, the stability of eNOS regulated by miR-455-3p and NO production is induced by H_2_S. Moreover, H_2_S levels and miR-455-3p are incremented in human atherosclerosis plaques, implying that H_2_S could be involved in the miR-455-3p/eNOS/NO pathway controlling atherosclerosis development [128].

Sirtuin-1 has been reported to prevent premature senescence of ECs, protecting from dysfunction [129]. Exogenous H_2_S directly induces sirtuin-1 sulfhydration and stability, reducing aortic inflammation and formation of atherosclerosis plaques [130].

Further studies demonstrated that H_2_S reduces the severity of atherosclerosis in a mouse model of disturbed blood-flow, through the upregulation of ACE-2 and increase in Ang(1–7) levels [131]. At cellular level, in LPS-activated ECs, H_2_S promotes the upregulation of the beneficial side of the renin-angiotensin system [131], documenting a multitargeting effect of H_2_S.

An original mechanism of action of H_2_S in controlling endothelial dysfunction in atherosclerosis was recently proposed [50]. In both cultured ECs and in mice, endogenous CSE-derived H_2_S leads to sulfhydration and dimerization of the RNA-binding protein human antigen R (HuR), described to be inhibited in atherosclerosis [50]. The administration of SG1002, a slow polysulfide donor, in ECs isolated from CSE knockout mice, re-established HuR sulfhydration with subsequent inflammatory marker (CD62E) downregulation. Moreover, SG1002, administered to ApoE^−/−^ CSE knockout mice exposed to partial carotid ligation, limited plaque formation, demonstrating an H_2_S-induced antiatherogenic effect [50].

Based on the above results, it appears that H_2_S donors may be a potential promise for the treatment of endothelial inflammation related disorders [132].

### 7.2. Proangiogenic Effect

Several studies report the effects of H_2_S, derived from endogenous biosynthesis or released by exogenous donors, on the process of angiogenesis and in the wound healing context, mainly at low micromolar concentration range, mimicking the physiological concentration of the gasotransmitter [15,68,105]. Additionally, CSE overexpression promotes in vitro angiogenesis [68,133], while CSE silencing, KO or pharmacological inhibition blocks in vitro and in vivo neovascularization responses [133,134]. In addition to the activation of the autocrine eNOS pathway [7], the exposure of ECs to VEGF produces an increase in CSE-dependent H_2_S [134].

Recently, a role of 3-MTS participation in angiogenesis occurrence has been demonstrated in vitro [135]. A connection between 3-MTS-derived H_2_S and EC metabolism has been demonstrated: 3-MTS downregulation decreased mitochondrial respiration and ATP production, increased glucose uptake, and perturbed the whole EC metabolome [135].

Pro-angiogenic effects of H_2_S are evident as increased EC proliferation, migration, and tube formation in vitro. Exogenous H_2_S has been shown also to promote in vivo angiogenesis in models of chicken chorioallantoic membrane and to induce neovascularization in mouse subcutaneous Matrigel plugs [15,134]. In a model of cutaneous burn injury and wound healing, topical administration of a H_2_S-saturated physiological solution has been demonstrated even to significantly increase the wound closure [134].

Therefore, various studies have investigated the cellular signaling pathways involved in the pro-angiogenic effect of H_2_S to discover its molecular targets (Figure 1). Hydrogen sulfide has been shown to activate multiple signaling pathways with a key role in the contribution of EC migration during angiogenesis. Exposure of ECs to H_2_S donors induced increased phosphorylation of Akt, ERK1/2, and p38 MAPK, resulting in their activation [15,134].

There is also evidence about the effect of H_2_S on the activity of eNOS, promoting its phosphorylation on Ser1177 and consequent NO production inside ECs [133,136,137]. Ultimately, H_2_S and NO emerged as being mutually dependent in inducing angiogenesis of ECs and vasorelaxation [68].

In addition, a reverse mechanism appeared to also be effective in controlling EC viability: NO, produced by eNOS, is able to induce CSE activation, resulting in further production of H_2_S in ECs [138].

However, the direct molecular target of H_2_S on angiogenesis remains to be elucidated. From studies of mass spectrometry and additional investigations, it emerged that a disulfide bond between Cys1045 and Cys1024 in the intracellular kinase core of VEGFR-2 serves as a molecular switch for H_2_S to regulate the function of VEGFR-2 [139]. In particular, data revealed that HS^−^ (in aqueous solution, H_2_S is a mixture of H_2_S and HS^−^) breaks an inhibitory disulfide bond, bringing VEGFR-2 in an active conformation, probably promoting the activation of downstream signaling [139,140].

An alternative theory about H_2_S interaction with its target molecules is associated with S-sulfhydration, a post-translational modification of cysteine residues, induced by H_2_S on target proteins, involved in signaling pathways [141]. An example of this mechanism is S-sulfhydration of eNOS on Cys443 by NaHS, resulting in increased activity and stability of eNOS and promotion of its phosphorylation, with higher NO bioavailability in ECs to promote their survival and trophism [69].

### 7.3. Wound Healing Promotion

H_2_S has been reported to accelerate the healing of gastric ulcers and skin burn wounds [15,134,142]. Topical application of H_2_S improved recovery from burns in wild-type rats, while genetic ablation of CSE delayed healing in mice [134]. H_2_S improves angiogenesis and wound healing in db/db mice by promoting transcription of VEGF, epidermal growth factor (EGF), HIF-1α and eNOS, by upregulating VEGF and platelet-derived growth factor (PDGF) proteins and receptor phosphorylation [143,144]. H_2_S accelerates wound healing in STZ-induced diabetic mice with the formation of granulation tissue and increased levels of anti-inflammatory factors and VEGF [59]. Additionally, attenuation of inflammation has been attributed to H_2_S, thus improving diabetic wound healing in ob/ob mice [111]. Accordingly, H_2_S facilitates wound closure through the inhibition of neutrophil extracellular traps (NET) release-coupled neutrophil death (NETosis) in db/db mice [114]. Interestingly, H_2_S improved wound healing via restoration of endothelial progenitor cell functions and activation of angiopoietin-1 in db/db mice [113].

Recent epigenetic data document that treatment of ECs with H_2_S or upregulation of CSE rescued migration impairment due to high glucose, through a pathway involving miR126-3p upregulation and DNA methyl trasferase-1 downregulation [145].

## 8. Therapeutic Strategies to Improve H_2_S Concentration at Endothelial Level

**Figure 2:**
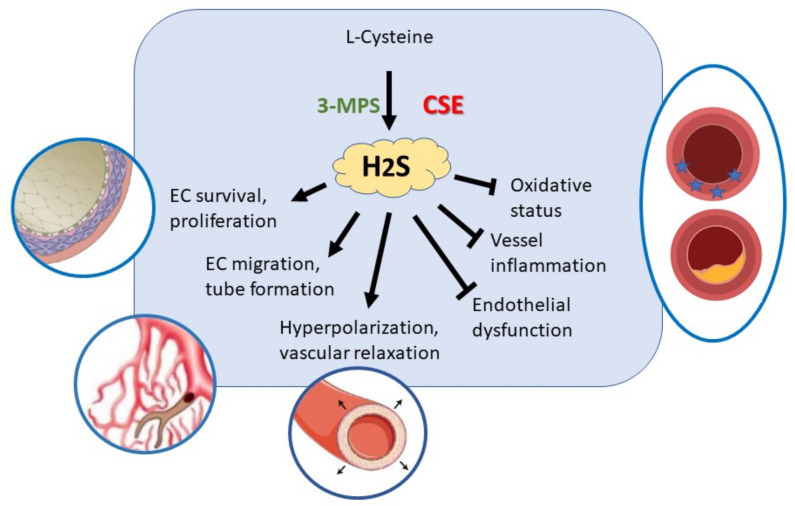
H_2_S exerts autocrine/paracrine actions in vascular endothelial cells in order to maintain their trophism and physiological functions.

The use of H_2_S donor compounds or gene therapy to increase the expression of enzymes responsible for the endogenous synthesis of H_2_S has the aim of restoring endothelial function and preventing the onset of pathologies associated with endothelial damage. Several efforts have been made to synthetize effective H_2_S donors showing different H_2_S releasing kinetics and site of action. The main objective has been to control blood pressure and correct endothelial dysfunction, vascular inflammation and redox state and to improve neovascularization and healing of wounds (Figure 2). Furthermore, H_2_S donor drugs have been evaluated as hypoglycemic agents in type 2 diabetes. H_2_S has been shown not only to protect cells from damage induced by hyperglycemia, but to prevent the onset of type 2 diabetes, preserving the functionality of β-pancreatic cells and regulating the sensitivity of target organs to insulin [146].

Due to the divergent responses induced by different concentrations of H_2_S, it is important for H_2_S donor drugs to maintain the plasma concentration of H_2_S at physiological levels, in the nanomolar order. Therefore, an ideal H_2_S donor should possess two qualities: slow and gradual production and intracellular release of H_2_S.

Indeed, NaHS, widely used for experimental purposes as H_2_S donor, is unsuitable for clinical use, due to its fast kinetics of H_2_S release, difficulties to titer the dosage, and its toxic effects [147,148].

A slow-releasing H_2_S donor was developed, GYY4137, demonstrating its vasodilating property in aortic, renal, and cardiac arteries in an L-NAME-induced hypertension model [85]. The antiatherogenic and endothelium-dependent vasodilating effects of GYY4137 were reported in ApoE^−/−^ mice, through decreasing vascular inflammation (lower ICAM-1, IL-6 and TNF-α expression) and oxidative stress [149]. An additional antithrombotic action was demonstrated in mice [150]. Among the others, GYY4137 was proposed in post-ischemia remodeling. The beneficial effects on cardiac functions were correlated to greater vessel density in the infarcted area [151].

The mitochondria-targeted H_2_S donors AP123 and AP39 have been demonstrated to prevent hyperglycemia induced oxidative stress and metabolic alteration in microvascular ECs, suggesting their use in vascular complications of diabetes [119]. The slow releasing H_2_S donor AP39 remarkably reduced systemic blood pressure, heart rate and arterial stiffness in L-NAME treated rats [88].

The positive interaction and synergistic action between NO and H_2_S [152] lead to the development of H_2_S-NO hybrid donor as ZYZ-803, recently reported to promote angiogenesis with a crosstalk between STAT-3 and CAMKII [153]. The authors reported an increased blood flow and vascular density in the hind limbs of mice exposed to femoral artery ligation.

The orally available prodrug SG1002 is an inorganic mixture (sodium polysulthionate) which in vivo was demonstrated to increase both H_2_S and NO levels [154]. Its protective effects have been demonstrated in animal models of atherosclerosis and acute limb ischemia, and patients with heart failure [50,132,155,156].

Among the established drugs, ACE inhibitors bearing a SH group, such as captopril, can promote blood pressure reduction though the sulfhydryl moiety beside the primary pharmacological target. Zofenopril demonstrates vasorelaxant and proangiogenic properties in addition to its ACE inhibitory activity. Indeed, we have contributed to demonstrating that its active moiety, zofenoprilat, can be considered an H_2_S donor and an upregulator of CSE expression at the EC level [80,157,158,159].

A further therapeutic option is represented by H_2_S-releasing derivatives of a number of drugs, such as non-steroidal anti-inflammatory drugs [160]. Alongside its antithrombotic properties, H_2_S releasing-aspirin was recently demonstrated to exert pro-proliferative and anti-apoptotic actions on cultured ECs together with anti-inflammatory and anti-oxidative features [161].

The use of orally active compounds able to endogenously produce H_2_S, such as *N*-acetylcysteine (NAC) and taurine, has been proposed, but clinical trials unfortunately were not followed up with published data (Table 1). NAC, a well-tolerated compound, clinically employed to enhance cellular levels of glutathione, is rapidly cleaved in vivo to yield cysteine. On the other hand, in vivo and ex vivo studies demonstrate that the sulfur amino acid taurine markedly and dose-dependently increased the expression of both CSE and CBS, with a higher effect on CSE upregulation [162]. A reduction in blood pressure in patients with prehypertension has been described [163].

In the complex, only very few clinical studies on H_2_S donors or enhancers administered in endothelial dysfunction-related disorders are currently registered in NIH ClinicalTrials.gov as listed in Table 1, but no results have been posted or are available on PubMed.

In a manner similar to NAC, cysteine/cysteine-rich undenatured whey protein supplement improved pressure ulcer recovery in a small group of diabetic patients [164].

The development of natural compounds, present in the diet, as a H_2_S source is interesting, such as polysulfides. Diallyl trisulfide (DATS), diallyl disulfide (DADS) and diallyl sulfide (DAS) are the active principles of the Alliaceae family, such as garlic, which is recognized worldwide as a popular remedy of hypertension. These polysulfides have been demonstrated to exert vasodilating properties in relation to H_2_S release [165], behaving as anti-hypertensives in L-NAME-treated rats [166]. In animal experiments, DATS improved cardiac function in aortic constricted mice, via an upregulation of VEGF, reduced angiostatin and increased myocardial vascular density [167]. Systemic administration of DATS or local transplantation of DATS-treated or CSE-overexpressing bone marrow cells improved capillary density, cell survival and blood perfusion in ischemic hindlimb of db/db mice [168]. Administration of DATS improved neovascularization in STZ-induced diabetic mice through increased NO availability [169].

Erucin [4-(methylthio) butyl isothiocyanate] is a natural isothiocyanate particularly abundant in Eruca sativa Mill. (rocket salad), an edible cruciferous plant belonging to the family of Brassicaceae. Isothiocyanates (ITCs) in general represent a source of different beneficial biological effects on human health, and most are investigated in relation to their chemo-preventive and anti-cancer properties [170,171,172]. Numerous studies demonstrated a general anti-inflammatory and antioxidant activity [173], together with protective properties for the cardiovascular system, where ITCs exhibit vasorelaxing and antihypertensive activity and a protective effect against endothelial dysfunction [174,175,176]. Several biological effects of ITCs may be associated with their ability to release H_2_S inside cells in a slow and long-lasting manner, leading to the definition of “smart H_2_S-donors” [175,177]. H_2_S release from ITCs occurs in a specific manner, depending on the presence of thiols, and it is particularly facilitated in the cell cytosol, where high concentrations of organic thiols, glutathione (in 1–10 mM range), and cysteine (in 30–200 μM range) are present [175,176]. Natural isothiocyanates, including erucin, may therefore represent a possible exogenous source of H_2_S, which, if gradually released, could mimic the physiological concentrations of the endogenous gasotransmitters. On the other hand, they can be the base for the design of synthetic H_2_S donor hybrids with antioxidant property and interesting pharmacological development [178].

Considering the requirement of dressings able to protect ulcers with high exudate levels and to promote wound healing (i.e., in diabetic patients), medicated dressings have been designed and developed. In particular, a functional sodium alginate dressing with H_2_S-releasing properties (SA/JK-1) was fabricated incorporating a pH-dependent donor, JK-1 molecule, into a sodium alginate sponge [179]. The resulting construct provided a moist healing protection able to continuously release H_2_S under acidic pH and absorbing exudate at the wound interface. In vitro, the construct was demonstrated to be biocompatible and effective in improving fibroblast migration and proliferation. When tested in animal model of full thickness dermal defect, SA/JK-1 promoted granulation tissue formation, angiogenesis, collagen deposition, and re-epithelization [179]. Overlapping results were demonstrated by the same group with hyaluronic acid hydrogels doped with H_2_S which was shown to induce M2 macrophage polarization [180]. Another example is represented by silk fibroin porous scaffold loaded with GYY4137, reported to facilitate in vitro bone cell trophism and angiogenesis [181]. These data demonstrate that H_2_S-medicated wound dressing/biomaterial may represent promising strategies for non-healing wounds or bone healing and regeneration. Extensive animal and clinical studies are, however, necessary for assessing their safety and validation.

## 9. Concluding Remarks

H_2_S is nowadays considered an important transmitter able to maintain vascular homeostasis. Most of its activities are due to autocrine/paracrine actions by ECs, with a fine control of its plasma concentrations. The availability of H_2_S depends on the activity of endothelial and other cells that express the key enzymes involved in the gasotransmitter release, the reactivity of H_2_S and its inactivation by redox systems and the efficacy of elimination reactions. On top of these, the production of H_2_S by the gut microbiota and intestinal epithelium is important to consider, due to the increasing recognition of circulating molecules coming from this source and finely controlling the cardiovascular system performance [96,182].

There is still demand for the availability of safe and effective synthetic H_2_S donors or enhancers, and natural products or nutraceuticals are helping to fulfil this demand. Indeed, few clinical trials based on H_2_S exogenous sources have been interrupted or have not published their results for unknown reasons.

Since endothelial dysfunction and inflammation continue to be the main causes of morbidity and mortality all over the world, knowledge of the molecular and biochemical mechanisms underlying cardiovascular pathologies and their complications is still required, as well as the definition of new treatment options to prevent endothelial dysfunction or revert cardiovascular disorders [183,184]. The recent pandemic evidenced this unresolved medical need [185].

What it is expected from novel molecules in order to be druggable is the exhibition of H_2_S levels near the physiological ones, and many compounds are actually druggable H_2_S-donors, but it seems that no clinical trials are currently running against endothelial dysfunction (Table 1). Sulfur compounds with natural origin represent helpful pharmaceutical/nutraceutical tools to be used in therapy or as a template for the ideation of advanced H_2_S-donor molecules with improved pharmacodynamic and/or pharmacokinetic properties [132,178].

Although experimental data clearly document a protective effect of H_2_S donors against endothelial dysfunction, further clinical studies are needed. To the best of our knowledge, there are no clearly active clinical trials on patients affected by pathologies due to endothelial dysfunction and treated with H_2_S donors. Even a modest improvement in endothelial function and viability would be a therapeutic success due to the lack of drugs against this diffuse condition predisposing to cardiovascular pathologies.

## Figures and Tables

**Table 1:** Clinical trials on endothelial dysfunction-related diseases with H_2_S donors/enhancers.

Identifier Year (Location)	Condition or Disease	Drug	Phase	Status Results
NCT01232257 2011 (The Netharlands)	HYPERTENSION (Chronic Kidney Disease, Chronic Kidney Failure, End Stage Kidney Disease, End Stage Renal Disease)	*N*-acetylcysteine (NAC)	Phase 3	Completed No results posted
NCT03179163 2020 (USA)	HYPERTENSION	Captopril	Phase 1/2	Recruiting
NCT03410537 2018 (China)	DIABETES TYPE 2 (Lower Extremity Artery Disease)	Taurine vs. Placebo	ND	Recruiting No results posted
None	ATHEROSCLEROSIS/ THROMBOSIS	-	-	-
None	ANGIOGENESIS/ WOUND HEALING	-	-	-

The search of clinical trials listed in ClinicalTrials.gov was performed combining the keywords: H_2_S, hydrogen sulfide, endothelial dysfunction and the disease listed in the second column. Other not recent studies performed on patients are reported in the text (see [154]).
